# Supplementary material for: Complexome profiling of the Chlamydomonas psb28 mutant reveals TEF5 as an early PSII assembly factor
Source: Plant Cell. 2025 Mar 17;37(6):koaf055. doi: 10.1093/plcell/koaf055 (PMC12164590; doi:10.1093/plcell/koaf055)
Supplement: koaf055_Supplementary_Data [file koaf055_supplementary_data.zip › psb28_interactive.zip!/plots/intensity/qualityPlots/peptides/NDA5_Cre16.g671000.t1.2.html]

### Cre16.g671000.t1.2

Trivial name: NDA5  
SeqCoverage [median]: 14.10  
Mass: 54.95 kDa  
  
 

| Sequence | MissedCleavage | Mass [Da] | wtIntensity | muIntensity | Score |
| --- | --- | --- | --- | --- | --- |
| GALETEPTLR | 0 | 1085.57 | 265.55 | 1392.57 | 96.03 |
| GNKPQITLVDQADR | 1 | 1553.82 | 0.00 | 795.22 | 38.03 |
| LGLEAAAALAGGSR | 0 | 1255.69 | 0.00 | 775.35 | 79.82 |
| VGGAGPEAEAAVAGAAAAEGK | 0 | 1752.86 | 0.00 | 2379.91 | 50.92 |
| VSYSLASDAGSAAGGAAR | 0 | 1609.77 | 0.00 | 531.00 | 81.49 |
|  |  |  | 265.5 | 5874.0 |  |
